# Successful Surgical Repair of Complete Duodenal Transection Caused by Horse Kick: A Case Report

**DOI:** 10.70352/scrj.cr.24-0059

**Published:** 2025-03-11

**Authors:** Yusuke Asai, Yusuke Tsunetoshi, Yuta Susa, Akiko Matsuzawa, Seiji Miyazaki, Yuki Itagaki, Hiroyuki Yamamoto, Kotaro Kimura, Hiroki Kushiya, Shoki Sato, Naoya Okada, Takumi Yamabuki, Kentaro Kato, Yoshihiro Kinoshita, Minoru Takada, Yoshiyasu Ambo, Fumitaka Nakamura

**Affiliations:** Department of Surgery, Teine Keijinkai Hospital, Sapporo, Hokkaido, Japan

**Keywords:** blunt duodenal trauma, surgical repair, horse kick injury

## Abstract

**INTRODUCTION:**

Horse kicks are a rare cause of injury and tend to cause severe complications such as visceral organ injury. Traumatic duodenal injuries are associated with high mortality rates. Furthermore, their reconstructive procedures vary widely and require appropriate on-the-spot judgment by the surgeon. We experienced a case of blunt abdominal trauma with a complete transection of the first portion of the duodenum caused by a horse kick without any associated lesions. A good postoperative course was achieved by trimming the pyloric part of the stomach and performing an end-to-end anastomosis between the gastric remnant and the duodenum, along with tube decompression and biliary drainage.

**CASE PRESENTATION:**

A woman in her 50s was kicked in the right upper quadrant of her abdomen by a horse and transported to a local hospital. Computed tomography revealed pneumoperitoneum and hematoma near the duodenum, discontinuity of the duodenal wall, and a poorly contrasted area in the pancreas head. The patient underwent emergent laparotomy 6h after the accident. The first portion of the duodenum was completely lacerated. No contamination around the pancreatic head or saponification of fat tissue was observed. Because the patient’s vital signs were stable and the condition of the damaged tissue was favorable, the transection was repaired with trimming of the pyloric part of the stomach and end-to-end anastomosis between the gastric remnant and the duodenum. Decompression, feeding and biliary drainage tubes were placed. The patient’s postoperative course was favorable and the patient was discharged on postoperative day 20 in a stable condition. At an outpatient visit 3 months postoperatively, the patient reported no abdominal pain or stenosis symptoms.

**CONCLUSIONS:**

We experienced a rare case of a single complete duodenal transection due to a horse kick. End-to-end anastomosis with tube decompression and biliary drainage was performed because the patient’s vital signs were stable, there was little contamination or contusion of the surrounding tissue, and it had not been >24h since the injury. The patient had a good course of treatment.

## Abbreviations

AAST-OIS
: American Association for the Surgery of Trauma-Organ Injury Severity Scoring System
AIS
: Abbreviated Injury Score
C-tube
: cystic duct tube
CT
: computed tomography
ERCP
: endoscopic retrograde cholangiopancreatography
MRCP
: magnetic resonance cholangiopancreatography
POD
: postoperative day

## INTRODUCTION

Horse kicks are a rare cause of injury and tend to cause severe complications, such as visceral organ injury.^1–3)^ Traumatic duodenal injuries are associated with high mortality rates. Previous reports have shown overall mortality rates of 4%–30%.^4–8)^ Furthermore, their reconstructive procedures vary widely and require appropriate on-the-spot judgment by the surgeon. We experienced a case of blunt abdominal trauma with a complete transection of the first portion of the duodenum caused by a horse kick without any associated lesions. A good postoperative course was achieved through duodenal end-to-end anastomosis, tube decompression, and biliary drainage.

## CASE PRESENTATION

**Fig. 1:**
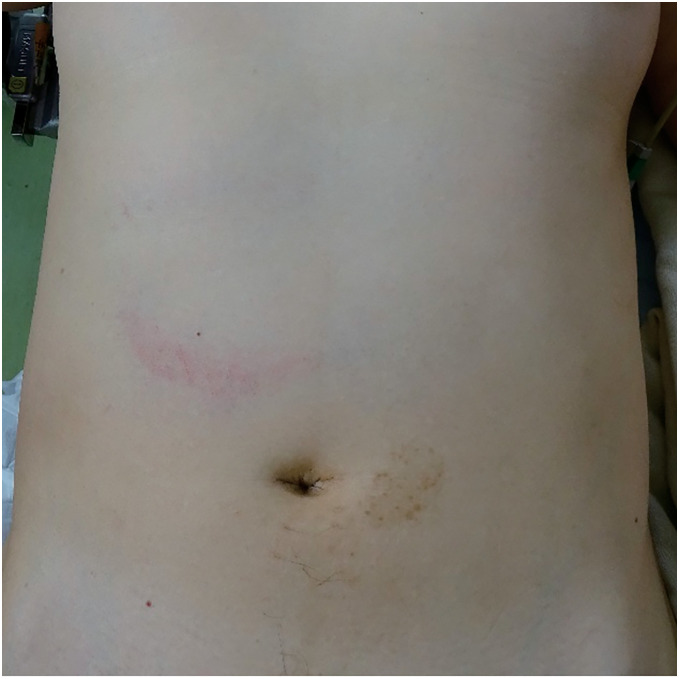
Physical findings. A bruise in the right upper quadrant of the abdomen.

**Fig. 2:**
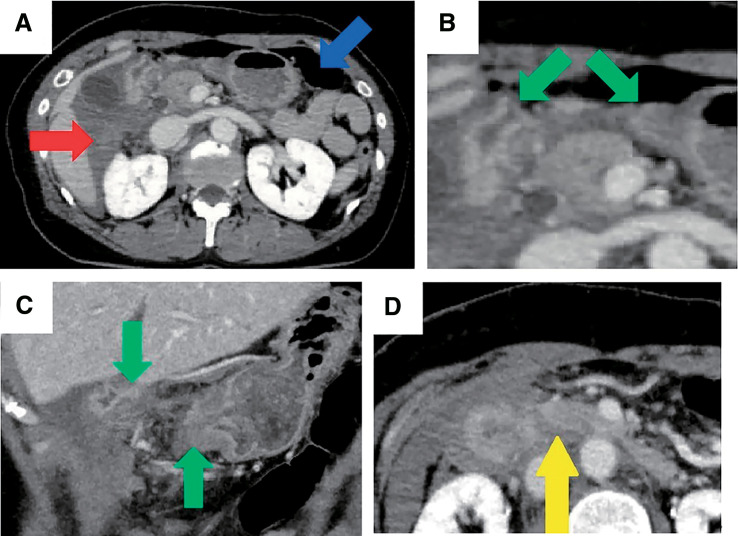
CT findings. (**A**) Red arrow indicates the intraperitoneal hematoma. Blue arrow indicates the pneumoperitoneum. (**B**, **C**) Green arrows indicate the lacerated duodenal stumps. (**D**) Yellow arrow indicates the poorly contrasted area in the pancreatic head. CT, computed tomography

A woman in her 50s was kicked in the right upper quadrant of her abdomen by a horse that weighed approximately 600 kg. The patient was initially transported to a local hospital. Pancreatic injury was suspected due to the nature of the injury, physical examinations, and contrast-enhanced computed tomography (CT), and the patient was referred to our hospital 3.5h after the accident. Upon arrival, the patient was alert with a blood pressure of 165/92 mmHg, heart rate of 82 bpm, SpO_2_ of 92% under room air, and body temperature of 37.4°C. Her abdomen had a bruise in the upper right quadrant with tenderness in the same area; however, no signs of peritoneal irritation were observed (**Fig. 1**). Blood test findings were as follows: white blood cell count, 8160/μL; hemoglobin, 11.7g/dL; platelets, 25.8×10^4^/μL; aspartate aminotransferase, 265 IU/L; alanine aminotransferase, 213IU/L; amylase, 247 IU/L; lipase, 408 IU/L; creatine phosphokinase, 180IU/L; and C-reactive protein, 0.03mg/dL. Repeat contrast-enhanced CT was performed after admission, which revealed pneumoperitoneum and hematoma near the duodenum, discontinuity of the duodenal wall, and a poorly contrasted area in the pancreas head (**Fig. 2**). Because duodenal laceration was suspected, the patient underwent emergent laparotomy 6h after the accident without additional examinations, such as magnetic resonance cholangiopancreatography (MRCP), to evaluate pancreatic duct injury, for the purpose of time-saving.

**Fig. 3:**
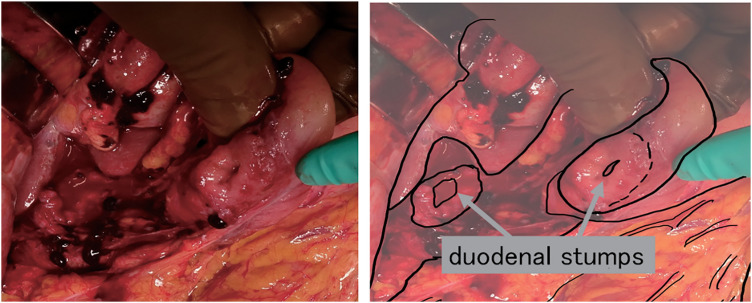
Intraoperative findings. The first portion of the duodenum was completely lacerated.

The operative findings were as follows: the first portion of the duodenum was completely lacerated (American Association for the Surgery of Trauma Organ Injury Severity Scoring System [AAST-OIS] Grade III; **Fig. 3**). An extended Kocher maneuver was performed, and the presence of any other organ injuries including the pancreatic head, was investigated. No contamination around the pancreatic head, saponification of fat tissue, or other injuries to major vessels and other organs were observed. We planned to evaluate the patient with intraoperative endoscopic retrograde cholangiopancreatography (ERCP) if any surgical findings suggested biliary or main pancreatic duct injury; however, no obvious injury to the pancreatic head was observed on intraoperative findings; therefore, intraoperative ERCP was omitted. Because the patient’s vital signs were stable and the condition of the damaged tissue was favorable, primary anastomosis was performed for repair.

**Fig. 4:**
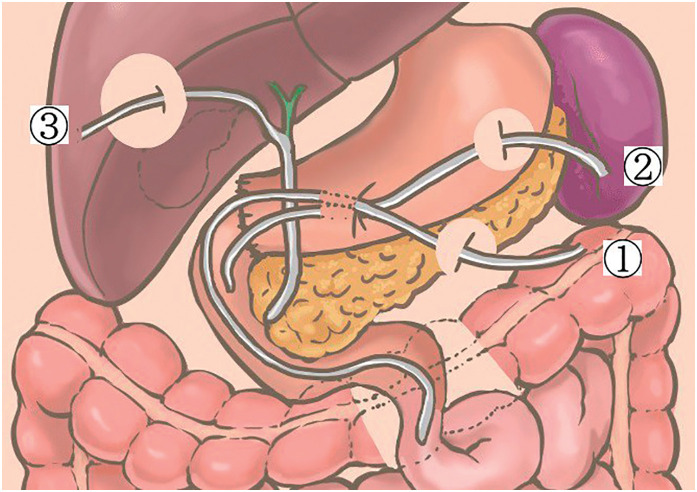
Schematic of the surgery. The transection was repaired with a hand-sewn end-to-end anastomosis. ① Feeding jejunostomy tube, ② duodenostomy tube, and ③ biliary drainage tube (C-tube) were placed. C-tube, cystic duct tube

The ruptured duodenum and pyloric portion of the stomach were trimmed and repaired by hand-sewn end-to-end anastomosis between the gastric remnant and duodenum using 4-0 Polydioxanone. Then, an antegrade decompression tube for gastric and duodenal juice drainage and a feeding jejunostomy tube were placed. Considering the possibility of bile congestion secondary to edema of the ampulla of Vater, delayed biliary injury, and anastomotic leakage, cholecystectomy was performed to place a C-tube (cystic duct tube) for biliary drainage as an adjunctive procedure (**Fig. 4**). Two drains were placed around the pancreas.

The patient’s postoperative course was favorable. The serum amylase and lipase levels were normalized on postoperative day (POD) 2. An oral contrast study on POD 8 revealed no leakage from the injury site and normal passage of contrast into the jejunum. A bile duct contrast study on POD 16 revealed normal passage of contrast into the duodenum. The decompression and feeding tubes were removed on POD 15, followed by the C-tube on POD 16. The patient was discharged on POD 20 in stable condition. At an outpatient visit 3 months postoperatively, the patient reported no abdominal pain or stenosis symptoms.

## DISCUSSION

Our case was one in which the isolated injury to the duodenum caused by a horse kick, which is a rare primary cause of injury, was treated with appropriate surgical interventions, including adjunctive procedures, leading to a good postoperative course.

Regarding horse-related injuries, fall-from-mount is the most common primary cause (54%–79%), followed by horse kicks (15%–22%).^9–11)^ Except for fall-from-mount injuries, facial (22.4%), abdominal (17.6%), and head injuries (15.3%) were the most common. Regarding abdominal injuries, the possibility of severe injury (Abbreviated Injury Score [AIS] 3) was high (60%).^9)^ The transfer of energy from the end of the hoof, which has a small cross-sectional area, to a small field, leads to internal organ injuries that are more severe than predicted.^3)^

Duodenal injuries account for 4.3% of all abdominal injuries. Moreover, 80% of duodenal injuries occur as a result of penetrating trauma, whereas the remaining 20% occur due to blunt trauma.^12)^ Duodenal injuries are most often accompanied by injuries to other organs due to their anatomic proximity to other organs: 86.9% of duodenal injuries have multiple associated injuries,^13)^ with the liver being the most commonly injured organ (17%). Other organs include the colon (13%), the pancreas (12%), the small intestine (11%), the stomach (9%), and major vessels (15%). Isolated duodenal injuries, as seen in this case, are rare. There are various mechanisms underlying blunt duodenal injury: (i) injury due to direct force, (ii) rupture due to increased internal pressure, and (iii) injury due to acceleration and deceleration forces. (i) A direct force is applied against the abdominal wall and transmitted to the duodenum, which is then projected against the spinal column. (ii) An external force compresses the stomach or duodenum and increases their internal pressure, causing the duodenal wall rupture. (iii) Acceleration and deceleration forces act on the mobile and nonmobile portions of the duodenum. In our case, no crushed tissues around the duodenal lacerated lesion were observed. Therefore, we speculate that the injury was caused by rupture due to increased internal pressure.

Although the mortality rate of duodenal injuries varies from 4% to 30%, the mortality rate of patients with complex duodenal trauma was 24%^4–7)^. An injury–operation delay of >24h increases the risk of postoperative duodenal fistula and death^7,14)^; therefore, early diagnosis and treatment of duodenal injuries are important. In this case, surgery was performed within 6h from the onset of injury, which may have contributed to the good outcome.

Whether preoperative MRCP should have been performed in this case is controversial. Preoperative ERCP was impossible because of the duodenal transection. Since MRCP is also a highly accurate test for main pancreatic duct injury,^15,16)^ preoperative MRCP imaging could be considered in this case. However, MRCP is a time-consuming test, and its use in this case, in which gastrointestinal perforation was evident, could have aggravated the situation. In addition, there is a report that MRCP failed to identify the main pancreatic duct injury,^17)^ so we believe that MRCP should not be overconfident.

Treatment strategies for duodenal injuries with AAST-OIS Grade 3 or higher are controversial. Adjunctive procedures may be required to protect the suture line in cases where a high risk of anastomotic leakage is anticipated because postoperative duodenal leaks are considered the main factor for increased morbidity and mortality.^18)^ There are several types of adjunctive procedures, such as “triple tube ostomy” and “duodenal diverticulization.” First, Stone and Fabian^19)^ introduced the use of a duodenostomy tube as “triple ostomy” (gastrostomy, duodenostomy, and jejunostomy). Their study included 237 patients, and only one patient had a duodenal fistula when the duodenostomy tube was used, compared with eight patients when it was not used. Next, Donovan and Hagen^20)^ proposed “duodenal diverticulization,” which included primary repair of the duodenal perforation, gastrojejunostomy, and oversewing the duodenal stump with an end duodenostomy. This procedure is familiar to abdominal surgeons because it resembles Billroth-II reconstruction after distal gastrectomy. Some authors have doubted the necessity of adjunctive procedures for protecting the primary duodenal repair.^21,22)^ There have been reports of good outcomes with primary repair even in duodenal injuries of AAST-OIS Grade 3.^8)^ AAST guidelines state that surgery for a single duodenal injury of AAST-OIS Grade 3 or higher is controversial and that good results have been obtained with primary repair, nasogastric tube decompression, and drain placement.^23)^ In contrast, Japan Advanced Trauma Evaluation and Care guidelines state that for injuries >50% of the total circumference, the risk of complications is so high that additional surgery or repair methods other than simple suture closure should be considered.^24)^

In our case, we repaired the ruptured duodenum using end-to-end anastomosis between the gastric remnant and duodenum and placed a decompression tube, feeding tube, and C-tube as adjunctive procedures. The end-to-end anastomosis, compared to pyloric exclusion or duodenal diverticulization, is technically simple and allows for easier postoperative ERCP. However, there is a risk of anastomotic leakage associated with this technique. Thus, it should be performed in low-risk cases, such as the one presented in this report, characterized by stable vital signs, minimal contamination and contusion of the surrounding tissue, and a time interval of less than 24h from the onset of injury to surgery. If the tissue surrounding the anastomosis is contused or there is obvious pancreatic injury and concern about postoperative pancreatic fistula, pyloric closure or duodenal diverticulization may be considered. The C-tube was inserted not only for the decompression of the duodenum but also for the prevention of biliary stasis, which may occur due to edema of the ampulla of Vater. Decompression and feeding tubes were placed to prevent anastomotic leakage and postoperative small bowel obstruction. A retrograde duodenostomy tube was not placed because it could easily become dislodged and thus be ineffective.

## CONCLUSIONS

We experienced a rare case of a single complete duodenal transection due to a horse kick. End-to-end anastomosis with tube decompression and biliary drainage was performed because the patient’s vital signs were stable, there was little contamination or contusion of the surrounding tissue, and it had not been >24h since the injury. The patient had a good course of treatment.

## ACKNOWLEDGMENTS

The authors would like to thank Makiko Shimazu, our hospital’s research staff, and Enago (www.enago.jp) for the English language review.

## DECLARATIONS

### Funding

There are no sponsors or funding sources for this work.

### Authors’ contributions

YAsai drafted the manuscript.

YAsai and MT performed the operation.

YT supervised the writing and provided important revisions.

All authors have read and approved the final manuscript.

All authors agree to be responsible for all aspects of this study.

### Availability of data and materials

Not applicable.

### Ethics approval and consent to participate

This work does not require ethical considerations or approval. No specific consent was obtained because individuals could not be identified from this report.

### Consent for publication

Consent was obtained from the patient for the publication of this case report and any accompanying images.

### Competing interests

The authors declare that they have no competing interests.
